# Improving Timeliness in Surgical Discharge Summary Distribution: A Quality Improvement Initiative

**DOI:** 10.1177/2473974X221134106

**Published:** 2022-10-25

**Authors:** Peng You, Jimmy Liu, Louise Moist, Kevin Fung, Julie E. Strychowsky

**Affiliations:** 1 Department of Otolaryngology–Head and Neck Surgery, Western University, London, Canada; 2 London Health Sciences Centre Quality and Performance, London, Canada; 3 Department of Medicine, Schulich School of Medicine, Western University, London, Canada

**Keywords:** PS/QI, transitions of care, discharge summaries

## Abstract

**Objective:**

To implement a quality improvement initiative to achieve an institutional targeted discharge summary distribution metric of 50% within 48 hours of patient discharge from hospital within an academic tertiary care otolaryngology–head and neck surgery department.

**Methods:**

A pre- and postintervention study was conducted. Process mapping was performed. Interventions included education and engagement, implementation of auto-authentication (distribution immediately following transcription without review by the most responsible physician), and audit and feedback. The percentage of discharge summaries dictated with the auto-authentication code was evaluated. Process measures were collected for 12 months pre- and postimplementation. Balancing measures included workload and revisions to auto-authenticated notes. Analysis included summary statistics, statistical process control charting, and unpaired *t* tests.

**Results:**

The mean ± SD percentage of discharge summaries distributed within 48 hours increased from 19% ± 6.4% preintervention to 54% ± 20% postintervention (*P* < .0001). Seventy-four percent of discharge summaries were dictated via the auto-authentication code. The target metric was met in 71% of discharges with the auto-authentication codes as compared with 26% with non–auto-authentication. The interventions did not result in any change to perceived workload, and the incidence of auto-authentication revisions was <1%. The results were sustained with an increase of 72% the following quarter. For fiscal year 2021-2022, performance remained sustained with an 85% completion rate.

**Discussion:**

Our surgical department exceeded and sustained the targeted metric for timely discharge summary distribution using a quality improvement approach.

**Implications for Practice:**

Timely distribution of discharge summaries optimizes patients’ transitions of care and can be achieved through stakeholder education and engagement, auto-authentication, and audit with feedback.

The transition from hospital to home is a critical point for patients. Discharge summaries are a written form of communication that contains a description of the hospital stay, diagnoses, interventions performed, and recommended follow-up plans. Timely and accurate transfer of information at the time of discharge between inpatient and outpatient physicians is crucial for patient safety and health care efficiency. Delays in the distribution of discharge summaries negatively affect the quality of care of primary care providers (PCPs) and contribute to their dissatisfaction.^1^ Furthermore, delay in the dissemination of discharge summaries leads to higher readmission rates.^2,3^

Health Quality Ontario has listed the improvement of discharge summary distribution within 48 hours as a priority performance indicator. Our Medical Advisory Committee and Local Health Integration Network set an institutional target to distribute at least 50% of discharge summaries within 48 hours of patient discharge from the hospital. Despite these benchmarks, only 33% of summaries were distributed within 48 hours, and the average time for discharge summary distribution was 265 hours for the preceding 1 year within our regional hospital.

In the otolaryngology–head and neck surgery (OHNS) department at the London Health Sciences Centre (LHSC; London, Canada), discharge summaries are generated through dictation services and connected to an electronic medical record system. When a patient is discharged, a discharge summary is dictated by a team member who is often a trainee (resident or fellow), transcribed, authenticated (reviewed for accuracy and signed off) by the most responsible physician, and then electronically distributed to the patient’s PCP.

We sought to improve timely discharge summary distribution within the OHNS department of an academic tertiary care medical center and achieve a 50% distribution rate of discharge summaries within 48 hours of patient discharge from the hospital by September 2019. This article aims to share our quality improvement (QI) methodologies and results following a QI framework.

## Methods

### Problem Characterization

**Figure 1.**
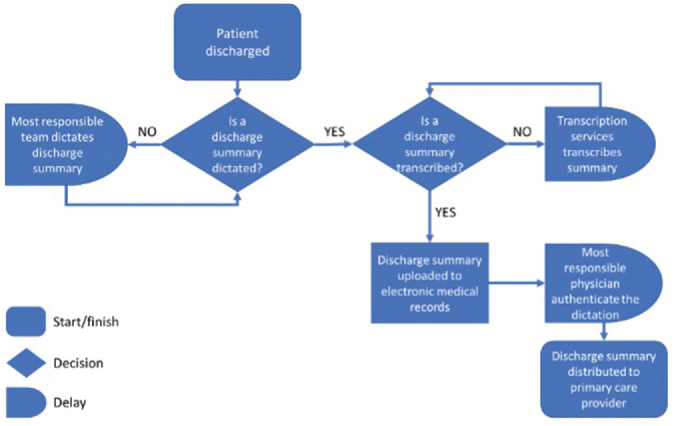
Process mapping areas of possible delay in the distribution of discharge summaries.

Process mapping and review of baseline performance data identified areas of possible delay in the distribution of discharge summaries (**Figure 1**). Review of process measures showed considerable delays between period of discharge and dictation, as well as transcription and authentication. On average, the time from discharge to authentication was 200 hours in the OHNS department vs 265 hours for the hospital for fiscal year 2017-2018. Additional data informed discharge to dictation (OHNS vs hospital: 84 vs 109 hours), dictation to transcription (17 vs 23 hours), and transcription to authentication (99 vs 133 hours).

### Stakeholder Involvement

Key stakeholders and multilevel champions were identified early, from leadership to house staff. This included members from the Medical Advisory Committee, Local Health Integration Network, Transcription Services, Health Information Management Services and Decision Support, and the Center for Quality, Innovation, and Safety, as well as consultants and resident physicians.

### Interventions

Given the problem characterization and review of baseline performance metrics, the stakeholder team determined potential change ideas. The QI initiative included 2 essential interventions. First, we encouraged the practice of same-day dictations through a department-wide education session. The information session aimed to inform consultants and residents of the rationale for timely discharge summary distribution and to present baseline data to promote engagement via audit and feedback. It also helped educate the group on the content necessary on a discharge summary. In addition, institutional and provincial targets for distribution were highlighted. Continuing education was provided at existing quarterly departmental meetings and served to identify any perceived barriers. During these meetings, the updated discharge summary performance measures were shared as audit and feedback.

Second, we implemented an auto-authentication dictation option to eliminate the existing delay from transcription to authentication. While in the past, the most responsible physician must manually authorize a discharge summary dictation before it is distributed, auto-authentication allows the distribution of the discharge summary immediately after transcription. The auto-authentication process was selected by the dictator using a different task code at the time of dictation.

In keeping with the frame of competency-based medical education, junior residents had their discharge summaries reviewed by the consultant for accuracy and completeness prior to the delegation for them to use the auto-authentication process. This served to ensure that the junior residents were well versed with the format and expected information within a discharge summary document.

### Project Design and Implementation Strategy

The Institute for Healthcare Improvement’s Model for Improvement was used to frame the QI strategy (**Table 1**), and a PDSA cycles approach (plan-do-study-act) helped to ensure that the interventions were having the intended effect and served to safeguard against unintentional consequences. After each PDSA cycle, the impact of the QI initiative was reviewed at a departmental meeting. Action plans were then formulated by the identified barriers. Education and engagement were implemented in July 2018 and auto-authentication 3 months later in September 2018.

**Table 1.** Institute for Healthcare Improvement’s Model for Improvement.

What are we trying to accomplish?	We aimed to distribute at least 50% of discharge summaries within 48 hours in the Department of Otolaryngology–Head and Neck Surgery
How will we know that a change is an improvement?	Data provided by Quality and Performance Department allowed comparison with baseline departmental results and hospital at large
What change can we make that will result in improvement?	• Consultant- and resident-level champions • Education and engagement • Auto-authentication of transcribed discharge summaries • Quarterly review of performance measures

The QI initiative was conducted as a pre- and postintervention study. The preintervention group comprised discharge summary data from the 12 months preceding the implementation of the first intervention (July 2017–June 2018). Performance metrics for the OHNS and the hospital as an additional comparator were used. The postintervention period included the 12 months following the implementation of the second intervention in September 2018. Performance data continue to be collected and disseminated, and updated data are included to demonstrate the sustainability of the interventions.

### Setting and Participants

The study was conducted at the OHNS department within the greater hospital. This study received ethics exemption by the Research Ethics Board at Western University (London, Canada). The reporting of this study is consistent with SQUIRE guidelines.

### Performance Measures and Evaluation

Process and balancing measures were considered. The primary performance measure, a process measure, was the percentage of discharge summaries distributed within 48 hours of patient discharge from the hospital. Additional process measures included time between key intervals: the time from patient discharge to discharge summary dictation, transcription, and distribution. To evaluate the effectiveness of implementation, the percentage of discharge summaries dictated with the novel auto-authentication code was evaluated and compared with the percentage of discharge summaries dictated with the original non–auto-authentication code. Monthly metrics were collected for 12 months following the implementation of the auto-authentication intervention. For comparison, hospital-wide performance data were collected over the same interval. Data were displayed by stoplight dashboard and statistical process control charts at quarterly meetings and communicated to additional stakeholders and executive leadership as audit and feedback. Pre- and postintervention statistical comparisons between groups were conducted by unpaired *t* tests with a level of significance of *P* < .05.

Balancing measures included feedback from residents and consultants, members of the most responsible team tasked with dictating the discharge summaries, and incidence of requested revisions. A quarterly anonymous online survey was distributed at the beginning of this QI project to measure the impact on workload. Consultant and resident participants were asked to reflect on their subjective rating of burden related to dictating discharge summaries for the preceding 3 months on a 5-point Likert scale (1, not burdensome; 5, very burdensome). General feedback about the QI project was collected in free-form text. The survey was then repeated for 6 months after the QI interventions. The incidence of addendums/revisions made to the discharge summaries was also tracked during the QI initiative.

## Results

**Figure 2.**
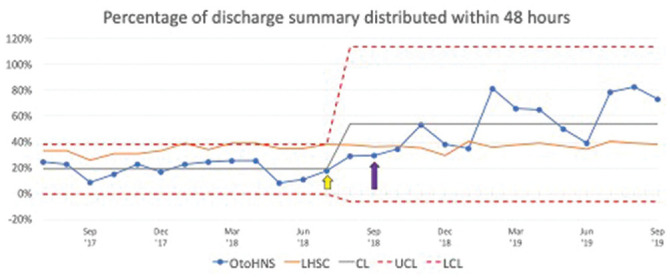
Statistical process control chart shows the percentage of discharge summary distributed within 48 hours. Yellow arrow, education intervention; purple arrow, auto-authentication intervention. CL, center line; LHSC, London Health Sciences Centre; LCL, lower control limit; OHNS, otolaryngology–head and neck surgery; UCL, upper control limit.

This QI initiative had a positive effect on the timeliness of discharge summary distribution. The percentage of discharge summaries distributed within 48 hours increased dramatically following the QI interventions (**Figure 2**). **Figure 2** also compares the performance of OHNS with LHSC-wide data for the study period. The LHSC-wide distribution rate was largely unchanged.

The mean ± SD percentage of dictations distributed within 48 hours of discharge increased from 19% ± 6.4% preintervention to 54% ± 20% postintervention (unpaired *t* test, *P* < .0001). Statistical process control charting suggested special cause variation after implementation by September 2019, 12 months after implementation of the auto-authentication code. These results were sustained with quarter 1 of financial year 2020-2021: 72% of discharge summaries were distributed within 48 hours of patient discharge from the hospital, as opposed to 55% for the hospital system at large. There was a modest increase following the initial education, engagement, and emphasis of same-day dictations. The addition of the auto-authentication option resulted in a convincing trend. Preintervention, 19% ± 6.4% of discharge summaries were distributed within 48 hours as compared with 54% ± 20% postintervention (*P* < .0001). Twelve months following the introduction of auto-authentication, 73% of discharge summaries were distributed within 48 hours, and the average time of discharge summary distribution was 60 hours. Comparatively, for the same month, the figures were 38% and 134 hours for our institution.

According to the statistical process control chart, a dip in percentage of discharge summaries distributed within 48 hours is evident from December 2018 to January 2019. During this time, there was a system-wide delay at transcription services; therefore, the time from dictation to transcription markedly increased. This had resolved by February 2019. Another dip was evident in June 2019. For that month, only 50% of discharge summaries were dictated with the auto-authentication code. June also represents the end of the academic year, and perhaps residents were less likely to make discharge summaries a priority.

The implementation of the intervention is evaluated by examining the process measure of percentage of discharge summaries dictated by using the auto-authentication code and comparing it with the percentage of discharge summaries dictated by using the original non–auto-authentication code. Seventy-four percent of discharge summaries over the study period were dictated with the auto-authentication code, with 71% distributed within 48 hours, as compared with 26% distributed within 48 hours with the non–auto-authentication code.

The QI initiative did not change the most responsible team’s perception of workload. Residents and consultants reported the burden of work in discharge summary dictations before our QI initiative to be 3.25 (n = 8, SD = 0.7) and 2.1 (n = 10, SD = 0.99), respectively. After 3 months, residents had a rating of 3.0 (n = 5, SD = 1.4) and consultants 2.2 (n = 10, SD = 0.92). The ratings were 3.13 (n = 8, SD = 1.55) and 1.13 (n = 8, SD = 0.35) for residents and consults after 6 months. The free-text feedback was overall positive to the adoption of the QI interventions.

Following the introduction of the QI initiative, 7 of 759 distributed discharge summaries had amendments as of March 2019. A review of the amendments showed the revisions to be minor details, such as spelling, with no major changes to care plans made. The updated versions with the amendments were automatically redistributed to the PCP. Given that <1% of dictations were revised, this balancing measure was not reviewed beyond March 2019.

## Discussion

Our department-led QI initiative improved the timely distribution of discharge summaries to surpass the targeted metric. This result was independent of other departments within our hospital, which showed minimal improvement. Delays in discharge summary distribution is a challenge faced by several health care systems.^1,4,5^ These delays mean that PCPs or other health care providers may be tasked to treat patients discharged from the hospital without a clear picture of the hospitalization, recent interventions, or intended care plans.^5^ The association between discharge summary availability and health outcomes such as readmission rates have led some authors to advocate this as a focal point for QI projects.^2,6^ Within surgery, the QI initiative tackling timely discharge summary distribution is sparse.^4^ This is the first QI initiative to tackle discharge summary distribution within OHNS.

Following our departmental QI interventions, the discharge-to-distribution time decreased dramatically, highlighting the success of the interventions. There were also marked decreases in times from discharge to dictation and from transcription to distribution. Furthermore, the proportion of discharge summaries distributed within the 48 hours following discharge increased markedly from 20% to 73%. In the 12 months following the initial introduction of auto-authentication, our department not only met the institutional objective of 50% discharge summaries distribution within 48 hours but also became the best-performing department within the greater hospital. A celebration of this success likely contributed to further engagement.

A careful review of the QI process with PDSA cycles following implementation of change ideas helped to minimize the risk of unintended effects. The incidence of amendments was used as a surrogate marker for the accuracy of the discharge summaries. Within our new model, residents and consultants still have ready access to the transcribed discharge summaries. Whenever an amendment is made, the updated version is automatically redistributed to the PCP. When these revisions were reviewed, the edits were minor and did not lead to changes to the patient’s care plan. Moreover, the anonymous surveys showed that the interventions were warmly received, and no notable change to the burden of work was reported. In other words, the QI interventions helped to facilitate communication among care providers during a critical transition period for patients without any notable increase in inaccuracies or impact on workload.

Some authors have sought to combat the delays in discharge summary distribution with innovative strategies such as electronic discharge summary programs.^4,7,8^ Through a retrospective study, Reinke et al found that the electronic discharge summary programs increased the timeliness with which the discharge summaries were completed. Similarly, Gilliam et al used an electronic discharge summary tool to ensure that all discharge summaries were completed by the time of first postdischarge clinical contact, as opposed to 43% before their QI intervention.

Working within the confines of our existing system, we approached this QI opportunity by first thinking about the hierarchy of effectiveness. Education and awareness are valuable but often not very effective. This can be appreciated in our QI initiative by the modest improvement after education. Nevertheless, education was essential to inform and engage stakeholders, as many consultants and residents were not aware of the importance of and existing target for discharge summary distribution. To complement the adaptive change following education, we also needed to incorporate elements of a system-oriented intervention such as automation. Auto-authentication was able to bypass the considerable delay between transcription and authentication in a systematic fashion. This strategy was relatively low resource intensive and did not require an overhaul of the existing process, such as adopting an electronic discharge summary program.

Contributors to our success can be best interpreted within Kotter’s model for change.^9^ A sense of urgency was created due to inadequate performance in distributing discharge summaries locally, especially as provincial bodies have identified this metric to be a priority indicator. Early stages for planning of this QI initiative served to build a guiding coalition by identifying stakeholders and multilevel champions. The design of the QI project also helped clarify the strategic vision. Moreover, our QI initiative purposefully included a quarterly presentation of the interventional outcomes to the department to continue stakeholder engagement. These meetings served as platforms to report and celebrate short-term wins. The last phase in Kotter’s model for change may perhaps be the most difficult: institutionalization. Cultural shifts are often slow, and our experience suggests that a combination of education and leadership support is essential. Moreover, ongoing iterative review of performance metrics are crucial to maintaining change, as the ongoing feedback allowed for accountability.

This QI project was completed in a surgical program. While our small department with 16 consultants and 16 residents has allowed for better engagement of stakeholders, it is also a limitation to be considered in extrapolating our findings to a larger and more heterogenous group. The setting of our QI initiative is within a surgical department where the discharge summaries may differ in complexity or focus than other disciplines. Success has been reported in a QI study involving internal medicine residents.^10^ Bischoff et al achieved improved timeliness of discharge summaries through careful QI planning, educational curriculum, an electronic discharge summary template, regular data feedback, and a financial incentive. Other factors to consider are that the ways for creating and distributing discharge summaries are not uniform across centers. These differences in logistics may create unique challenges at the local level that would need to be addressed.

Several challenges were encountered. The original auto-authentication code (code 53) worked at only 2 of 3 hospital sites; this code was changed to code 10 on November 28, 2018. Therefore, pilot testing is important to consider so that the fidelity of the intervention can be assessed. We can consider this intervention in our small academic department as a pilot test prior to consideration of expanding implementation throughout the rest of our hospital system. An additional barrier was that time from dictation to transcription was prolonged in December 2018 due to a hospital-wide transcription services delay that month.

It is important to consider the effectiveness of the implementation of the auto-authentication intervention as well. Despite the availability of the auto-authentication code, some consultants and residents continued to use the non–auto-authentication code (code 33). For example, in quarter 3 in fiscal year 2019-2020, 42% of discharge summaries were distributed within 48 hours (code 33 or code 10), but when we considered discharge summaries dictated only with the auto-authentication code (code 10), 62% were distributed within 48 hours. Change ideas to overcome this barrier should be considered. Given that this auto-authentication is currently being used solely in our department, when residents rotate through other departments, they are still using the non–auto-authentication code; therefore, use of the non–auto-authentication code may be more of a habit. As other departments adopt this same process, we may see an increase in the use of the auto-authentication code in our department. Another consideration is to remove the ability to use the non–auto-authentication code. Given that it is a hospital-wide dictation system, unless the entire system decides to remove the non–auto-authentication option, it will likely continue to exist.

Despite the limitations, we believe that the results of our study are encouraging. It showed that a well-constructed QI strategy could change critical metrics within patient care in a relatively short period. By engaging the learners from the very beginning of this QI process and adopting a continued PDSA approach, we are optimistic that the resulting cultural shift will make the outcome sustainable. The lessons that we learned and the auto-authentication process that we piloted from this QI project have been summarized, documented, standardized, and shared across the hospital system to promote hospital-wide QI on this indicator. As of July 2020, several other departments started adopting this new auto-authentication process. For fiscal year 2021-2022, performance in OHNS remained sustained with an 85% completion rate. Comparatively, the hospital-wide rate during this same time frame was 60%.

## Implications for Practice

In conclusion, we describe herein a QI initiative that successfully resulted in improving the timeliness of surgical discharge summary distribution without a notable impact on workload or error rate. The adoption of key stakeholders through education and the use of a system-oriented change of auto-authentication were the key to our success. Ultimately, our structured QI approach led to practice changes and improved a critical quality indicator. While this was a departmental QI project, it serves as a pivotal pilot project for transformative change at the organizational level.

## Author Contributions

**Peng You**, design, conduct, analysis, presentation of research; **Jimmy Liu**, conduct, analysis; **Louise Moist**, design, conduct, analysis; **Kevin Fung**, design, conduct, analysis; **Julie E. Strychowsky**, design, conduct, analysis, presentation of research

## Disclosures

**Competing interests:** None.

**Sponsorships:** None.

**Funding source:** none.
